# Loop Closure Detection Based on Residual Network and Capsule Network for Mobile Robot

**DOI:** 10.3390/s22197137

**Published:** 2022-09-21

**Authors:** Xin Zhang, Liaomo Zheng, Zhenhua Tan, Suo Li

**Affiliations:** 1 School of Mechanical Engineering, Shenyang Ligong University, Shenyang 110159, China; 2 Shenyang Institute of Computing Technology Co., Ltd., Chinese Academy of Sciences, Shenyang 110168, China; 3 Software College, Northeastern University, Shenyang 110169, China

**Keywords:** simultaneous localization and mapping (SLAM), mobile robot, loop closure detection, residual network (ResNet), capsule network (CapsNet)

## Abstract

Loop closure detection based on a residual network (ResNet) and a capsule network (CapsNet) is proposed to address the problems of low accuracy and poor robustness for mobile robot simultaneous localization and mapping (SLAM) in complex scenes. First, the residual network of a feature coding strategy is introduced to extract the shallow geometric features and deep semantic features of images, reduce the amount of image noise information, accelerate the convergence speed of the model, and solve the problems of gradient disappearance and network degradation of deep neural networks. Then, the dynamic routing mechanism of the capsule network is optimized through the entropy peak density, and a vector is used to represent the spatial position relationship between features, which can improve the ability of image feature extraction and expression to optimize the overall performance of networks. Finally, the optimized residual network and capsule network are fused to retain the differences and correlations between features, and the global feature descriptors and feature vectors are combined to calculate the similarity of image features for loop closure detection. The experimental results show that the proposed method can achieve loop closure detection for mobile robots in complex scenes, such as view changes, illumination changes, and dynamic objects, and improve the accuracy and robustness of mobile robot SLAM.

## 1. Introduction

Simultaneous localization and mapping (SLAM) is a mobile robot equipped with sensors, which constructs environmental maps by observing unknown environments and realizes simultaneous autonomous localization and navigation [1,2]. SLAM is widely applied in the autonomous navigation of mobile robots, virtual reality, smart homes, and other fields [3,4]. Due to the low cost of visual sensors that can obtain rich scene information, visual SLAM has attracted extensive attention [5]. Loop closure detection is an important part of SLAM, which plays an important role in reducing the accumulated error generated by the visual odometer, improving the accuracy of robot pose estimation, and constructing the global consistency map [6]. With the wide application of SLAM, the problems of low accuracy and the poor robustness of loop closure detection in complex scenarios need to be solved urgently [7].

Loop closure detection has been extensively studied by scholars [8]. Bag of Visual Words (BoVW) is a traditional method to achieve loop closure detection. To represent an image using the BoVW model, an image can be treated as a document. Similarly, “words” in images also need to be defined. Achieving this usually includes three steps: (1) feature extraction, (2) codebook construction, and (3) vector quantization [9,10]. It extracts artificial features such as the scale-invariant feature transform algorithm (SIFT) [11], the speed up robust features algorithm (SURF) [12], and oriented FAST and rotated BRIEF (ORB) [13]. Loop closure detection is realized by measuring the similarity of images [14]. Global characteristics information of a scene (GIST) adopts a two-dimensional filtering method to process regional texture information, extracts the overall features of the image, and improves the efficiency of loop closure detection [15]. However, traditional features are sensitive to environmental changes such as illumination changes, view changes, occlusion, dynamic objects, and a large scale, which affect the accuracy and robustness of loop closure detection [16,17].

Deep learning does not require the manual design of features and has strong feature extraction capability and good robustness to environmental changes. Therefore, deep learning is widely used in face recognition, scene classification, medical diagnosis, and other fields. Hou et al. [18] use deep learning to realize loop closure detection, which improves the accuracy of loop closure detection in illumination change scenarios compared with the BoVW and GIST methods. Sunderhauf et al. [19] used the AlexNet network to extract image features, which showed that mid-level features could better cope with changes in scene appearance and perspective. The Visual Geometry Group of Oxford University proposed the VGG network, which replaced AlexNet’s convolution kernel with a continuous convolution kernel, enhanced network performance by increasing network depth, and achieved excellent results [20].

Traditional deep learning methods can extract image features autonomously, but shallow features struggle to accurately describe the rich information of the image, and the spatial details of the image are ignored. The deepening of the number of network layers enlarges the storage space and increases the computation of traditional deep learning methods. The maximum pooling layer of traditional deep learning methods cannot represent the spatial location relationship between features and loses image detail information. Since the input and output of neurons are scalars, traditional deep learning models have a weak ability to represent image features [21].

Wang et al. [22] designed a deep learning model that combines the advantages of ResNet and CapsNet for improving the original structure to effectively classify remotely sensed lidar data. The structure of the ResNet is modified based on ResNet-34, and the outputs of ResNet are sent to CapsNet for lidar classification. Xiang et al. [23] propose a 3-D tumor computer-aided diagnosis (CADx) system with U-net and a residual-capsule neural network (Res-CapsNet) for ABUS images and provide a reference for early tumor diagnosis, especially non-mass lesions. Jampour et al. [24] presented a regularized CapsNet conjugated with ResNet-18 for signature identification. CapsNet allowed a powerful understanding of the objects’ components and their positions, while ResNet provided efficient feature extraction and description. The existing Res-CapsNet is applied in the fields of LiDAR data classification, image classification, and signature identification.

Loop closure detection based on a residual network and a capsule network (Res-CapsNet) is proposed to address the problems of low accuracy and poor robustness for mobile robot SLAM in complex scenes such as view changes, illumination changes, weather changes, and dynamic objects. It can improve the accuracy and robustness of the loop closure detection of the SLAM system and realize the autonomous positioning and navigation of mobile robots in complex scenes.

We combine the advantages of ResNet and CapsNet to design Res-CapNet for mobile robot SLAM. The main contributions of this paper are as follows: (1) A pre-trained ResNet model is used as a feature extractor to extract the shallow geometric features and deep semantic features of the image. The residual mechanism and GhostVLAD feature coding method are combined to obtain the global feature descriptors of the image. The GhostVLAD feature coding method can reduce the noise information in the image data and accelerate the convergence speed of the training model. (2) Dynamic routing is optimized by the entropy density peak, and the relative positions and directions between image features are extracted by CapsNet. The parameters are simple and robust to optimize the overall performance of the network. (3) Global feature descriptors and feature vectors are combined to contain the relative location distributions of features, retain the differences and correlations between features, and improve the accuracy of the SLAM system. Finally, in order to verify the feasibility of the proposed method, loop closure detection and SLAM experiments are designed, and the results are analyzed. The experimental results show that the proposed method is effective and robust.

The paper is organized as follows: In Section 2, we briefly discuss the deep convolutional neural network framework. Section 3 describes the novel architecture of the Res-CapsNet in detail. The experimental results and analysis are discussed in Section 4. Finally, the paper is concluded in Section 5.

## 2. Related Work

A deep convolutional neural network has the characteristics of local area perception, the up-sampling of the time domain, and weight sharing, which can make great breakthroughs in the recognition and classification of speech, text, image, and video. Network layer deepening enhances the network’s learning ability but reduces the convergence speed of the network. Gradient back propagation makes the gradient become infinitesimal, which makes it impossible to effectively adjust the weight of the network. It is difficult to realize reverse gradient conduction, resulting in gradient explosion, gradient disappearance, and large calculations.

In order to solve the problems of gradient disappearance and the network degradation of deep convolutional neural networks, He et al. [25] proposed a residual network (ResNet). It has a simple skip structure and a strong feature extraction capability, which is widely used in face recognition, automatic driving, and image classification. ResNet introduces a residual mechanism and adopts identity mapping to construct a residual unit, which reduces the number of network parameters and the computational complexity, improves the operational efficiency, solves the problem of network degradation, and improves network performance [26]. ResNet includes typical network structures such as ResNet-18, ResNet-34, ResNet-50, ResNet-101, and ResNet-152. Among them, ResNet-18 and ResNet-34 are composed of basic residual modules, and ResNet-50, ResNet-101, and ResNet-152 are composed of bottleneck modules.

**Figure 1:**
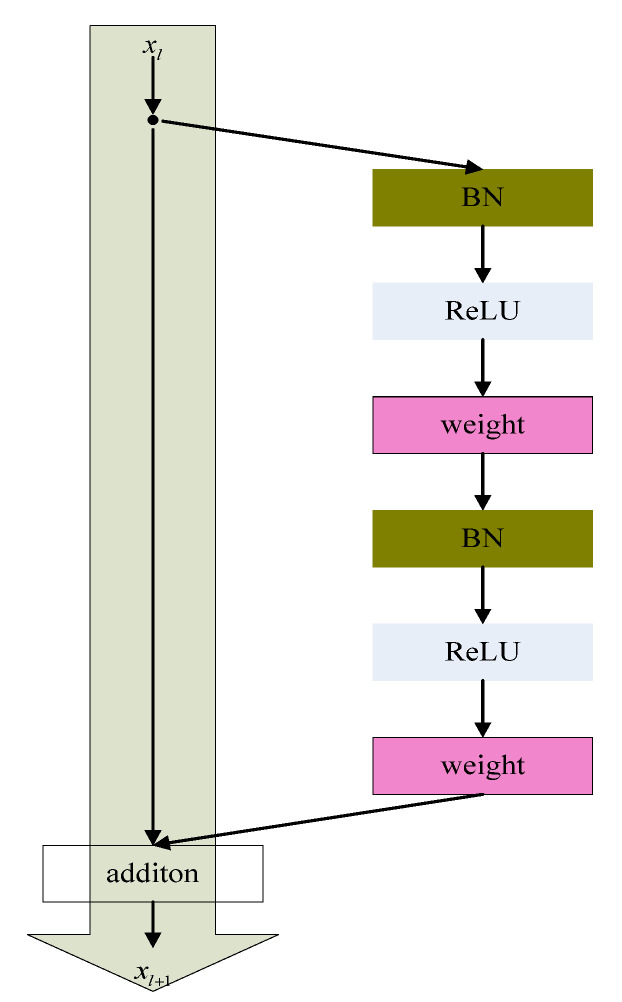
ResNetv2 [27].

The basic structure of ResNetv2 can be seen in Figure 1. ResNetv2 is composed of a weight, batch normalization (BN), and a nonlinear activation function (ReLU). Assuming that the input of the residual unit l, is $x_{l}$, then the output is:

(1) $$x_{l+1}=f\left(x_{l}+F\left(x_{l},W_{l}\right)\right)$$

where $F\left(x_{l},W_{l}\right)$ is the residual function, the residual function consists of two or three convolution layers, $W_{l}$ is the weight coefficient corresponding to the residual function, and $f\left(\cdot \right)$ is the nonlinear activation function that matches $x_{l}$ and $F\left(x_{l},W_{l}\right)$ to the same dimension by performing linear mapping of $W_{s}$.

ResNetv2 model uses a pre-activation mode in backward and forward propagation to make the information propagate faster, allowing the network to obtain better results, and this structure effectively prevents the gradient disappearance problem. Therefore, ResNetv2 was used in this paper.

## 3. Proposed Method

In order to improve the extraction and expression ability of image features, avoid the loss of spatial location features, improve the accuracy and robustness of loop closure detection, and realize the autonomous localization and mapping of mobile robots, in this paper a loop closure detection algorithm (Res-CapsNet) combining a deep residual network (ResNet) and a capsule network (CapsNet) is proposed.

### 3.1. Residual Network Model Based on Feature Coding Strategy

To solve the problems of gradient disappearance, network degradation, and the large amount of computation of deep neural networks and to speed up model convergence in training and meet the real-time requirements of a SLAM system, a residual network model based on a feature coding strategy is proposed in this paper.

**Figure 2:**
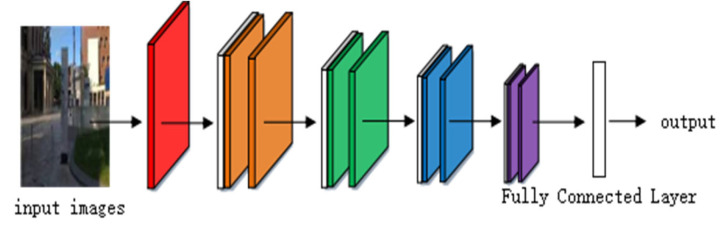
The structure of ResNet-50.

**Figure 3:**
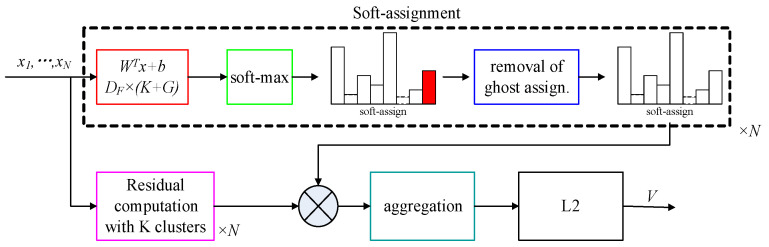
The algorithm flowchart of GhostVLAD.

Considering the number of model parameters and the training effect comprehensively, the ResNet-50 model is adopted as the basic network of feature extraction, as shown in Figure 2. This model is used to extract shallow geometric features and deep semantic features. Feature coding improves the recognition ability of ResNet by clustering the extracted image features. A vector of locally aggregated descriptors (VLAD) calculates the difference vectors of image feature descriptors and their clustering centers and aggregates local features into global features, which can solve the problem of image retrieval and image classification [28]. Arandjelović et al. [29] obtained global feature descriptions by clustering local features and extracting distribution relations among the features and proposed a VLAD coding algorithm, NetVLAD, combined with a neural network. Compared with the VLAD algorithm, this algorithm is more flexible and suitable for similar scene recognition. In order to extract high-quality image feature descriptors, Arandjelovi et al. [30] proposed the GhostVLAD algorithm by combining NetVLAD and “Ghost” central points, as shown in Figure 3.

Given N D-dimensional local image descriptors $\left\{X_{i}\right\}$ as inputs and *K* cluster centers $\left\{C_{k}\right\}$ as VLAD parameters, the output of VLAD is a D×K dimensional matrix, V. The element of $V\left(j,k\right)$ is computed as follows:

(2) $$V\left(j,k\right)=\sum_{i=1}^{N}\left(x_{i}\left(j\right)-c_{k}\left(j\right)\right)$$

where $x_{i}\left(j\right)$ is the *j*-th dimension of the *i*-th descriptor, and $c_{k}\left(j\right)$ is the *j*-th dimension of the *k*-th cluster center.

Due to the different amounts of information contained in the local feature descriptors of each cluster center, we set the weight parameter, $a_{k}\left(x_{i}\right)$ as the weight of $\left(x_{i}\left(j\right)-c_{k}\left(j\right)\right)$, which can describe the relationship between the local feature descriptors of each class:

(3) $$V\left(j,k\right)=\sum_{i=1}^{N}a_{k}\left(x_{i}\right)\left(x_{i}\left(j\right)-c_{k}\left(j\right)\right)$$

Soft assignments $\bar{a_{k}}\left(x_{i}\right)$ are replaced with:

(4) $$\bar{a_{k}}\left(x_{i}\right)=\frac{e^{w_{k}^{T}x_{i}+b_{k}}}{\sum_{k^{\prime }}e^{w_{k^{\prime }}^{T}x_{i}+b_{k^{\prime }}}}$$

Then, the global descriptor is:

(5) $$V\left(j,k\right)=\sum_{i=1}^{N}\frac{e^{w_{k}^{T}x_{i}+b_{k}}}{\sum_{k^{\prime }}e^{w_{k^{\prime }}^{T}x_{i}+b_{k^{\prime }}}}\left(x_{i}\left(j\right)-c_{k}\left(j\right)\right)$$

GhostVLAD is a global descriptor that describes the appearance of input images by adding a ghost clustering center and reduces the weight of low-quality images by automatic weighting. GhostVLAD is a generalization of NetVLAD, as with G=0 the two are equivalent.

**Figure 4:**
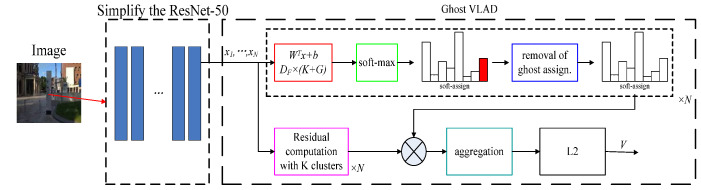
Network structure of ResNet based on GhostVLAD.

The input of the ResNet model is the color image of the real scene, and the image size is 224 × 224 × 3. The last mean pooling layer and full connection layer of ResNet-50 are removed as shown in Figure 4. The GhostVLAD layer is introduced, which distributes noisy information to the ghost classes to reduce the interference effect of noisy data. By training on the fused ResNet network and the GhostVLAD module, the GhostVLAD layer is dimensionally reduced to obtain a 512-dimension output vector, which can reduce the computational burden and effectively improve the robustness of scene recognition.

### 3.2. Peak Entropy Density Optimization of Capsule Network

In order to improve the accuracy of convolutional neural networks (CNN) image recognition and retain the spatial position relationship between image features, Hinton et al. [31] proposed the capsule network (CapsNet) for the first time in 2017. The dynamic routing mechanism of CapsNet adopts k-means clustering, which is only suitable for processing spherical data and is sensitive to the initial cluster center. Dynamic routing can be viewed as a parallel attention mechanism that allows each capsule at one level to attend to some active capsules at the level below and to ignore the others. This should allow the model to recognize multiple objects in the image, even if the objects overlap [22].

The optimal truncation distance is solved by optimizing the minimum value of entropy in this paper, and the dynamic routing optimized by density peak is adopted to improve the overall performance of CapsNet. Sabour et al. proposed CapsNet to improve the limitations of CNN feature extraction. By updating the dynamic routing mechanism between the master capsule and the digital capsule, high-level entity representation is obtained, which not only reduces network parameters but also avoids over-fitting. Through the experimental verification of MNIST datasets, compared with CNN, CapsNet has higher classification accuracy in digital recognition, traffic sign recognition, and medical image analysis [32,33,34].

**Figure 5:**
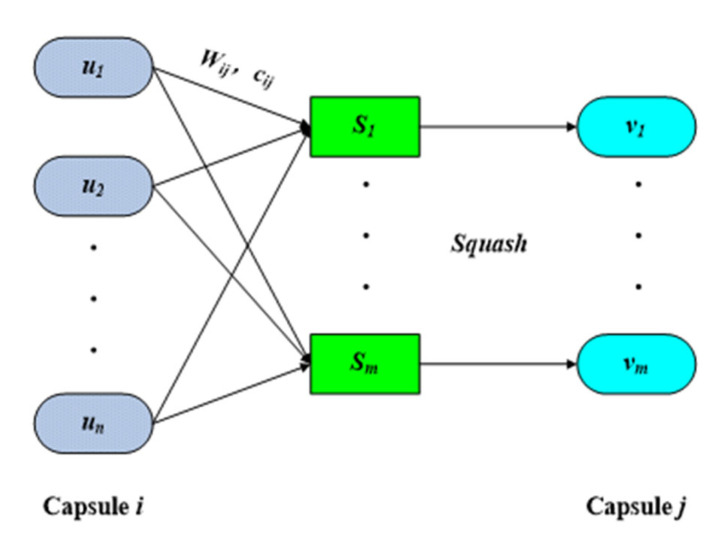
Information transmission between capsules [31].

The CapsNet structure is made of a network of capsules, which are used to represent image features instead of neurons in CNN [35]. Each capsule is a collection of neurons, and multiple capsules make up the entire capsule network. Each capsule represents all or part of the entity, the length of the vector represents the probability of the entity’s existence, and the direction of the vector represents various attributes of the entity in the image, such as posture (position, size, and direction), texture, deformation, and color. Dynamic routing is used to replace the maximum or average pooling layer; the output of each capsule is a vector, not a scalar; and vectorized capsules are used to encode feature information [36]. The information transmission process between capsules is shown in Figure 5.

A capsule is the basic operational unit of a capsule network, and each capsule is a collection of neurons. The input vector, $s_{i}$, is nonlinearly compressed through capsule i, and the capsule feature vector, $v_{i}$, is output as:

(6) $$v_{i}=\frac{{\left\|s_{i}\right\|}^{2}}{1+{\left\|s_{i}\right\|}^{2}}\frac{s_{i}}{\left\|s_{i}\right\|}$$

The length of the capsule’s feature vector, $v_{i}$, represents the probability of the existence of the target. The dimensional values represent the various attributes of the entity.

First, the capsule feature vector, $u_{j}$, of the upper layer in the network is multiplied by the weight matrix, $W_{ij}$, to generate the intermediate vector, ${\overset{⌢}{u}}_{i\left|j\right.}$:

(7) $${\overset{⌢}{u}}_{i\left|j\right.}=W_{ij}u_{j}$$

Then, the weighted sum of the intermediate vector, ${\overset{⌢}{u}}_{i\left|j\right.}$, is used to calculate the input vector, $s_{i}$:

(8) $$s_{i}=\sum_{j}c_{ij}{\overset{⌢}{u}}_{i\left|j\right.}$$

$b_{ij}$ is the coupling probability of capsule i and capsule j, and $b_{ij}$ is initially set to zero. The dynamic routing process of CapsNet is the updating process of weighted coefficient $c_{ij}$:

(9) $$c_{ij}=\frac{\exp\left(b_{ij}\right)}{\sum_{i}\exp\left(b_{ij}\right)}$$

Finally, the connection between adjacent capsules is completed to obtain the connection between low-level targets and high-level targets to realize the transmission and expression of characteristic information.

**Figure 6:**
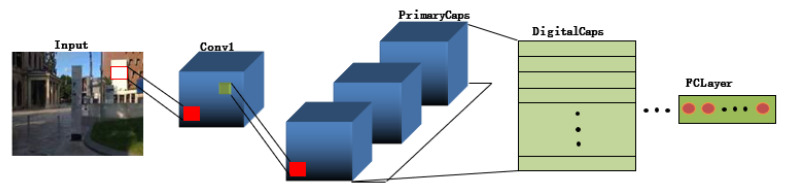
The structure of CapsNet.

Classical CapsNet consists of an input layer, convolutional layer (Conv1), initial capsule layer (PrimaryCaps), digital capsule layer (DigitalCaps), full connection layer, and output layer, as shown in Figure 6. Compared with the pooling strategy of CNN, the information transfer mechanism of CapsNet fully preserves the spatial position relation between features and realizes the accurate transmission of image information. The weighting coefficient, $c_{ij}$, is determined by the inner product between the prediction vector, ${\overset{⌢}{u}}_{i\left|j\right.}$, and the upper capsule, $v_{i}$.

The larger the inner product, the larger the weighted coefficient between the capsule neurons, indicating that the lower capsule transmits more characteristic information to the higher capsule. The smaller the inner product, the smaller the weighted coefficient between the capsule neurons, indicating that the lower capsule transmits less characteristic information to the higher capsule. Experiments available through three iterations can improve the coupling coefficient and will not increase the amount of calculation.

The dynamic routing mechanism of the capsule network adopts the k-means clustering algorithm to transform low-level features into high-level features that are only suitable for processing spherical data and are sensitive to the initial clustering center. Rodriguez et al. [37] proposed the density peaks clustering (DPC) algorithm in Science, which is suitable for arbitrary shape data with simple parameters and strong robustness. The dynamic routing mechanism of the capsule network is optimized by the density peak in this paper, and the optimal truncation distance is solved by optimizing the minimum entropy. The sensitivity of the capsule network to the initial cluster center is solved, and the aggregation of low-level features to high-level features is realized. The vector is used to represent the relative positions and directions between features to improve the overall performance of the network.

DPC mainly includes the local density, $\rho_{i}$, and the adjacent distance, $\delta_{i}$. A Gaussian kernel is used to define the local density as:

(10) $$\rho_{i}=\sum_{j}\exp\left(-{\left(\frac{d_{ij}}{d_{c}}\right)}^{2}\right)$$

The proximity distance is the minimum distance between data point $x_{i}$ and a point with a higher density, which can be expressed as:

(11) $$\delta_{i}=\left\{\begin{array}{c} \underset{j:\rho_{j}>\rho_{i}}{\min}\left\{d_{ij}\right\}\\ \max\left\{d_{ij}\right\} \end{array}\right.$$

Entropy is adopted to optimize truncation distance, and entropy is defined as:

(12) $$H=-\sum_{i=1}^{n}\frac{\rho_{i}}{Z}\log\left(\frac{\rho_{i}}{Z}\right)$$

(13) $$Z=\sum_{i=1}^{n}\rho_{i}$$

where Z is the standardization coefficient. By substituting Equation (10) into Equations (12) and (13), the function of the truncation distance is constructed, and the optimal truncation distance is solved by optimizing the minimum entropy. Experiments show that when entropy is minimal, the optimal value of the truncation distance, $d_{c}$, can be obtained. The specific optimization process is as follows:

Step 1. Realize the weight mapping of low-level capsules;

Step 2. The entropy is introduced to determine the truncation distance, $d_{c}$;

Step 3. Calculate the local density, $\rho_{i}$, and proximity distance, $\delta_{i}$;

Step 4. Calculate the connection probability between capsules according to the formula $c_{ij}=soft\max\left(b_{ij}\right)$;

Step 5. Calculate the total input, $s_{j}$, of the next capsule;

Step 6. Compress $s_{j}$ to $\left[0,1\right]$, update $b_{ij}$, and return $v_{j}$.

### 3.3. Fusion of the Optimized Residual Network and Capsule

**Figure 7:**
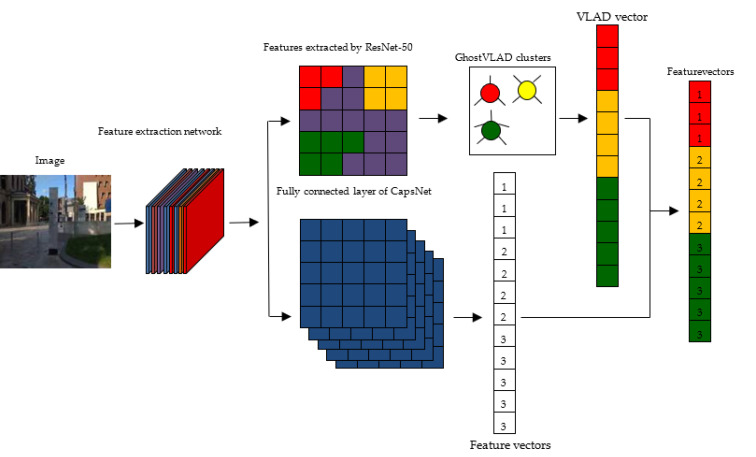
The feature vectors based on the capsule network and the residual network.

The residual network features are input into the GhostVLAD layer to obtain the sum of the residual errors of the feature points and clustering centers. The global feature descriptor is obtained by integrating the optimized features. Feature vectors representing feature distribution are obtained by the capsule network, then differentiated features are extracted, and global feature descriptors are combined with feature vectors. ResNet-50 features correspond to the abstract red, yellow, and green blocks in Figure 7. The full connection layer of CapsNet corresponds to the blue block and feature vector in Figure 7. The fusion of the two features includes the relative location distribution between the features, retains the difference and relevance between the features, and improves the accuracy of SLAM system localization and mapping.

**Figure 8:**
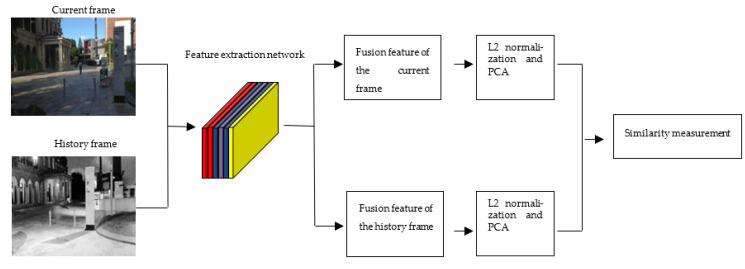
The flowchart of loop closure detection.

L2 normalization and principal component analysis (PCA) are used to reduce the dimensionality of the fused features, as shown in Figure 8. Then, the similarity of the image features is measured to determine whether a closed loop is formed. Res-CapsNet eliminates redundant image features and noise in the data, which not only improves the computational efficiency but also significantly improves the image expression ability, effectively establishes the environmentally consistent map, and improves the accuracy and robustness of SLAM system localization and mapping.

## 4. Experimental Results and Analysis

In order to verify the feasibility of the proposed method (Res-CapsNet), the standard SLAM datasets Gardens Point and TUM were used to evaluate the performance of our approach. Experimental platform: 8G memory and 3.5 GHz CPU.

In this application, the time cost was related to the size of the input data and the training epochs. For training the network, we used KITTI dataset [38] sequences 0–4 with dataset augmentation (approximately 100,000 images). Sequences 9 and 10 were used for validation. We kept a batch size of 5, as higher batch sizes resulted in bigger input tensors and, thus, were difficult to fit in GPU memory.

### 4.1. Evaluation Index

1. Precision and recall are commonly used indexes to evaluate the effectiveness of loop closure detection. The horizontal axis is the recall rate, the vertical axis is the precision, and the precision–recall curve is used to evaluate the effectiveness of algorithm.
  (14) $$precision=\frac{TP}{TP+FP}$$
  (15) $$recall=\frac{TP}{TP+FN}$$
  where TP represents the correct number of closed loops, FP represents the number of closed loops for error detection, and FN represents the number of true closed loops that are not detected.
2. The area under the curve (AUC) is the main index to evaluate loop closure detection. The closer the AUC value is to 1, the higher the average accuracy of the algorithm.
3. The absolute trajectory error (ATE) is the difference between the estimated trajectory and the real trajectory, which is the main index to evaluate the localization accuracy of SLAM.

### 4.2. Experimental Results and Analysis of Loop Closure Detection

Gardens Point dataset: the dataset was collected on the campus of Queensland University of Technology, including view changes, illumination changes, dynamic objects, and occlusion factors [39]. An image sample is shown in Table 1. The datasets were composed of three image subsequences. The image subsequences day-left and day-right were collected from the scenes on the left and right sides of the road during the day. Image subsequence night-right was collected from the scene on the right side of the same road at night.

**Figure 9:**
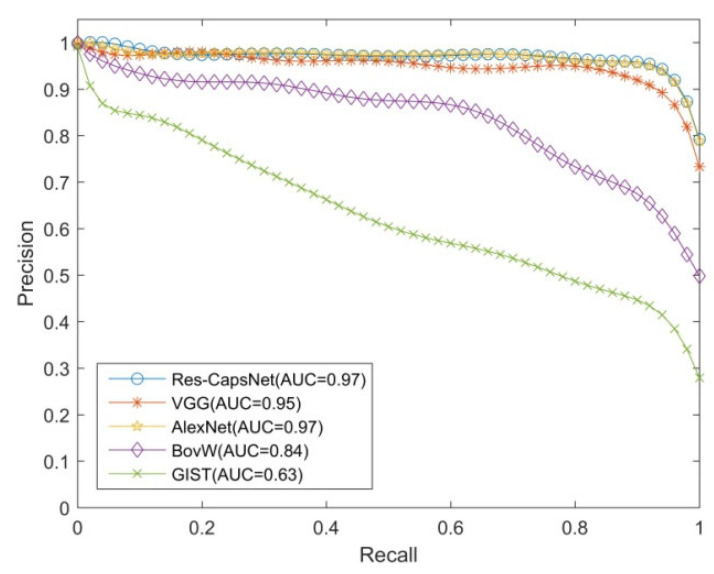
Precision–recall curves of the day-left vs. day-right datasets.

**Figure 10:**
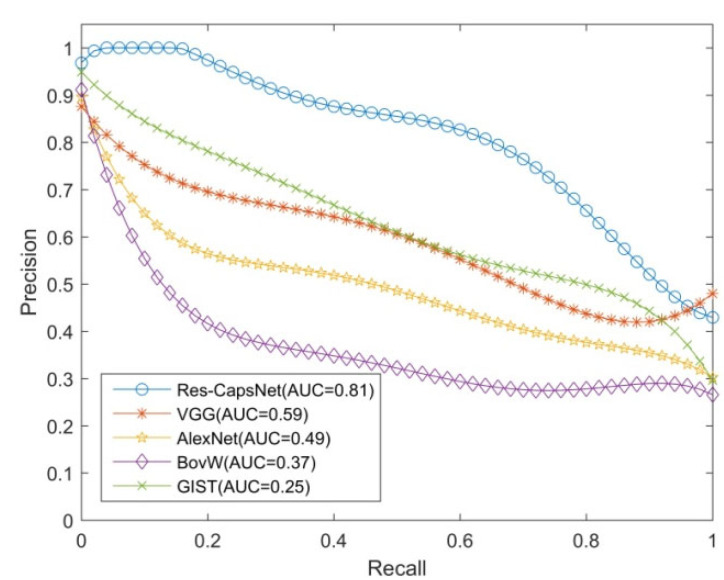
Precision–recall curves of the day-right vs. night-right datasets.

**Figure 11:**
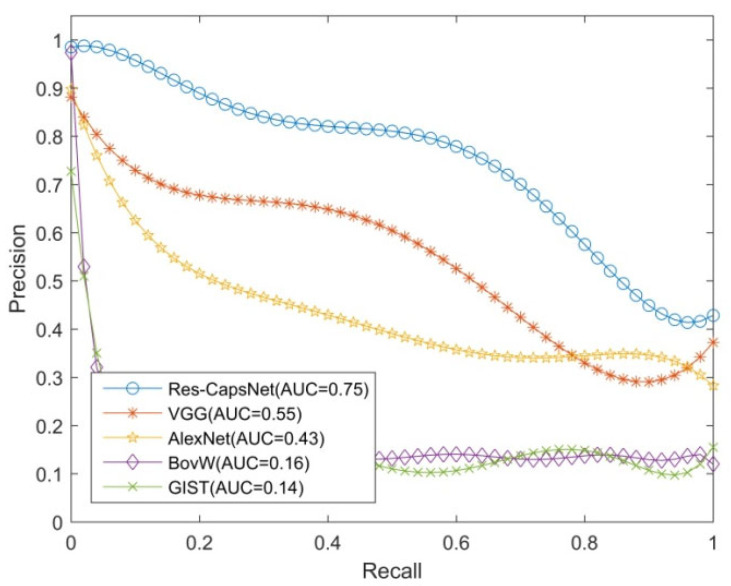
Precision–recall curves of day-left vs. night-right datasets.

The loop closure detection experiments were performed on the Gardens Point dataset to verify the effectiveness of the proposed method (Res-CapsNet). The dataset contains scene changes such as view, illumination, dynamic objects, and occlusion. The Res-CapsNet method in this paper was compared with the loop closure detection methods based on BoVW, GIST, AlexNet, and VGG. The experimental results are shown in Figure 9, Figure 10 and Figure 11, where the purple lines denote the visual bag model (BoVW), the red lines denote the loop closure detection based on GIST, the green lines denote the loop closure detection based on AlexNet, and the orange lines denote the loop closure detection based on VGG. The blue lines denote the Res-CapsNet method in this paper.

Figure 9 shows the loop closure detection experimental results of Gardens Point dataset under the same illumination with a changing perspective, testing the robustness of the proposed method for changing perspective. The AUC of the loop closure detection precision–recall curve based on Res-CapsNet was 0.97, and the average accuracy was also the highest. The AUC values for loop closure detection based on VGG, AlexNet, BoVW, and GIST were 0.95, 0.97, 0.84, and 0.63, respectively. When the recall rate was 80%, the precision of loop closure detection based on Res-CapsNet was 96.49%, while the precision values based on VGG, AlexNet, BoVW, and GIST were 94.97%, 96.18%, 73.28%, and 48.69%, respectively, in the scene with a changing perspective. The effects of loop closure detection based on VGG and AlexNet were similar, and the precision was higher than the loop closure detection based on BoVW and GIST, indicating that feature extraction based on the convolutional neural network model has good robustness for scenes with changing perspectives. The loop closure detection method based on Res-CapsNet maintained a high precision under a high recall rate.

Figure 10 shows the loop closure detection experimental results of the Gardens Point dataset with the same perspective and changing illumination, testing the robustness of the proposed method for changing illumination. The AUC of the loop closure detection precision–recall curve based on Res-CapsNet was 0.81, and the average accuracy was also the highest. The AUC values for loop closure detection based on VGG, AlexNet, BoVW, and GIST were 0.59, 0.49, 0.37 and 0.25, respectively. With the increase in the recall rate, the precision rate decreased gradually. When the recall rate was 80%, the accuracy of the Res-CapsNet method was 65.55%, while the closed-loop detection accuracy values based on VGG, AlexNet, BoVW, and GIST were 43.73%, 37.64%, 27.80%, and 49.89%, respectively. Res-CapsNet had a high precision under a high recall rate.

Figure 11 shows the loop closure detection experimental results of the Gardens Point dataset, which were used to test the robustness of the proposed method in a scenario with illumination changes and view changes. With the illumination changes and view changes of the environment, the performance values of all methods were degraded. The AUC of the loop closure detection precision–recall curve based on Res-CapsNet was 0.75, and the average accuracy was also the highest. The AUC values for loop closure detection based on VGG, AlexNet, BoVW, and GIST were 0.55, 0.43, 0.16 and 0.14, respectively. When the recall rate was 80%, the precision of loop closure detection based on Res-CapsNet was 57.53%, while the precision values based on VGG, AlexNet, BoVW, and GIST were 32.99%, 34.47%, 13.72% and 14.84%, respectively, in the scene with illumination changes and view changes. BoVW and GIST were less robust, suggesting that traditional features are susceptible to changes in illumination and perspective. Due to the image features extracted by the convolutional neural network, spatial details are lost. Therefore, the accuracy values of the AlexNet and VGG closed-loop detection methods were not greatly improved. Under the condition of a high recall rate, the loop closure detection precision based on Res-CapsNet was the highest.

**Figure 12:**
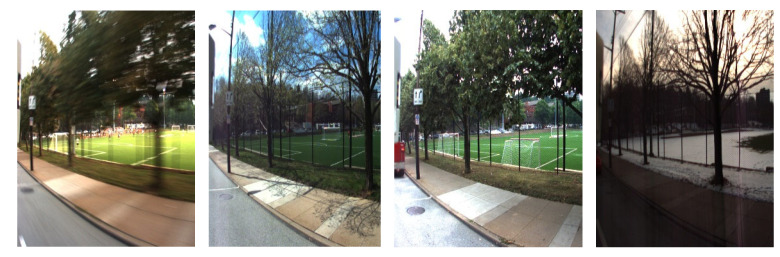
The same place during different seasons of a year from the CMU dataset.

The CMU visual localization dataset consists of multiple visual image sequences [40]. The image sequence was acquired by two monocular cameras mounted on a car. The car drove along the same route in Pittsburgh in different seasons. The image sequences belong to spring, summer, autumn, and winter, respectively, including light, weather, green vegetation, and visual changes produced by dynamic objects, as shown in Figure 12.

The CMU visual localization dataset contains seasonal, weather, light, green vegetation, and visual changes produced by dynamic objects for contrast experiments.

**Figure 13:**
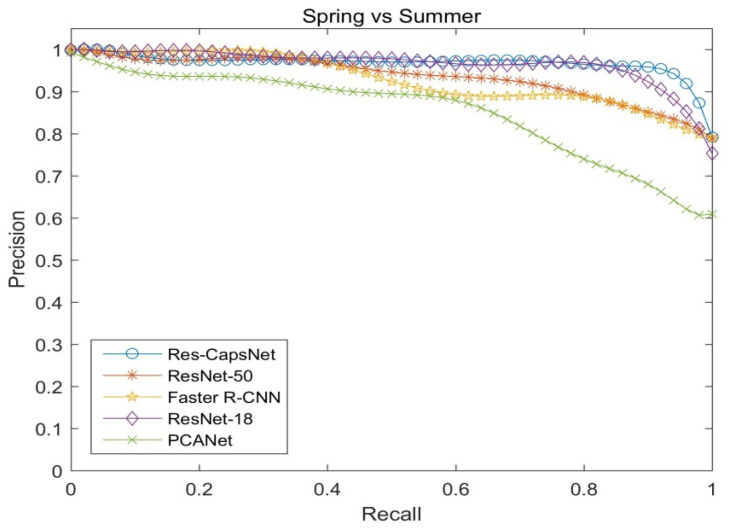
Precision–recall curves of four seasons comparison (spring vs. summer).

**Figure 14:**
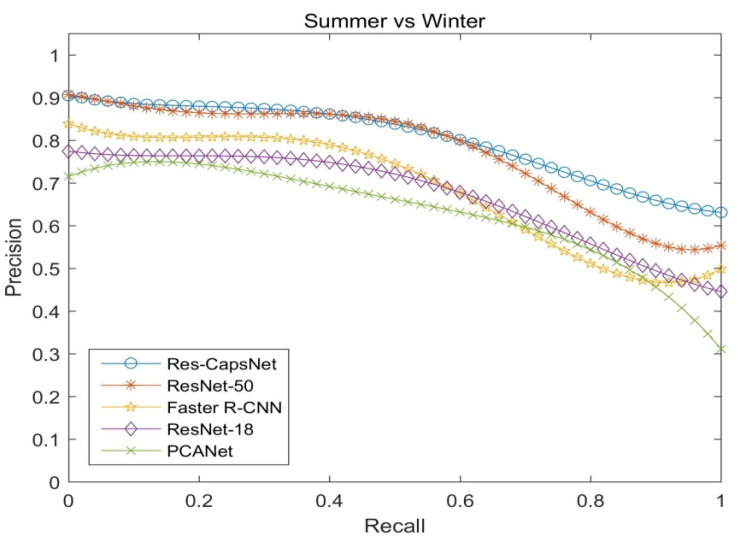
Precision–recall curves of four seasons comparison (summer vs. winter).

Figure 13 shows the precision–recall curves of the four seasons for the CMU visual localization dataset (spring vs. summer) by PCANet, ResNet-18, ResNet-50, Faster R-CNN, and Res-CapsNet, reflecting the impact of different seasons on loop closure detection. Figure 14 shows the precision–recall curves of the four seasons for the CMU visual localization dataset (summer vs. winter). The weather conditions vary from season to season. Since snow increases the difficulty of identification, summer and autumn are the easiest to match, and autumn and winter are the most difficult to match. Res-CapsNet can maintain a high recall rate with a high precision, and it has better robustness for visual place changes such as seasons.

### 4.3. SLAM System Experimental Results and Analysis

The TUM dataset was collected indoors at the Technical University of Munich, Germany [41]. The dataset was collected by Kinect, including an RGB color map and a depth map, image size 640 × 480, and the real pose trajectory file of the camera. The datasets contain dynamic and large-scale scenes that are targeted to motion blur, rotation, structure, texture, and loop closure situations to meet different testing needs. The parameters are shown in Table 2.

The “sitting” image sequence contains small movements of the human body, which is a low-dynamic scene. The “walking” image sequence contains pedestrians walking dynamically, which is a highly dynamic scene. The “office” image sequence contains the office scene with a track of more than 18 m, which belongs to the large-scale scene. The image was preprocessed, and the color image size was compressed to 224 × 224 × 3 by the scaling function as the input of the feature extraction network (ResNet50), where 224 was the image size and 3 referred to the three RGB channels. The proposed algorithm was compared with the classical ORB SLAM2, and the absolute trajectory error (ATE) was used to evaluate the accuracy of the SLAM system.

**Figure 15:**
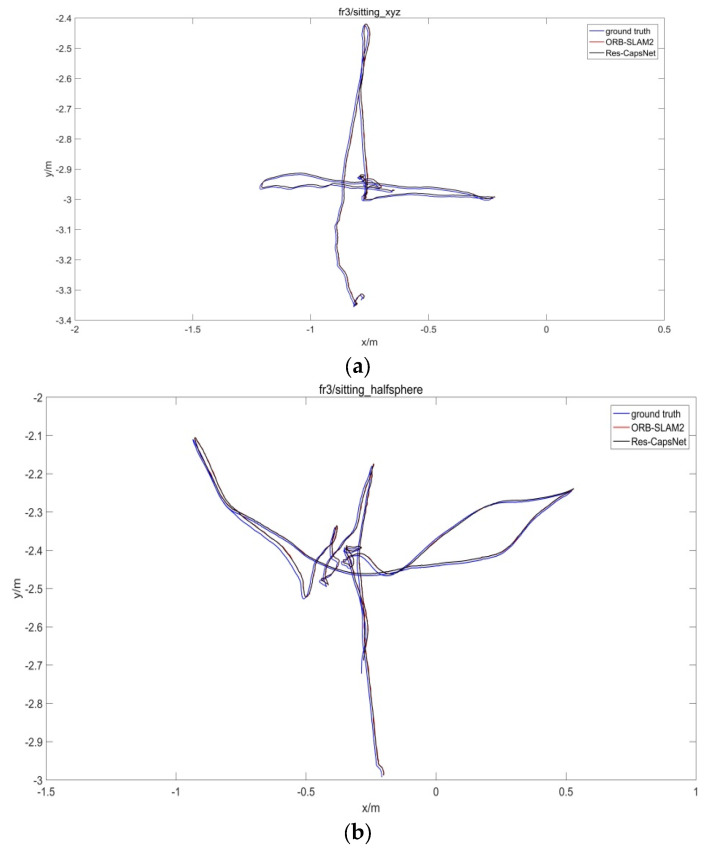

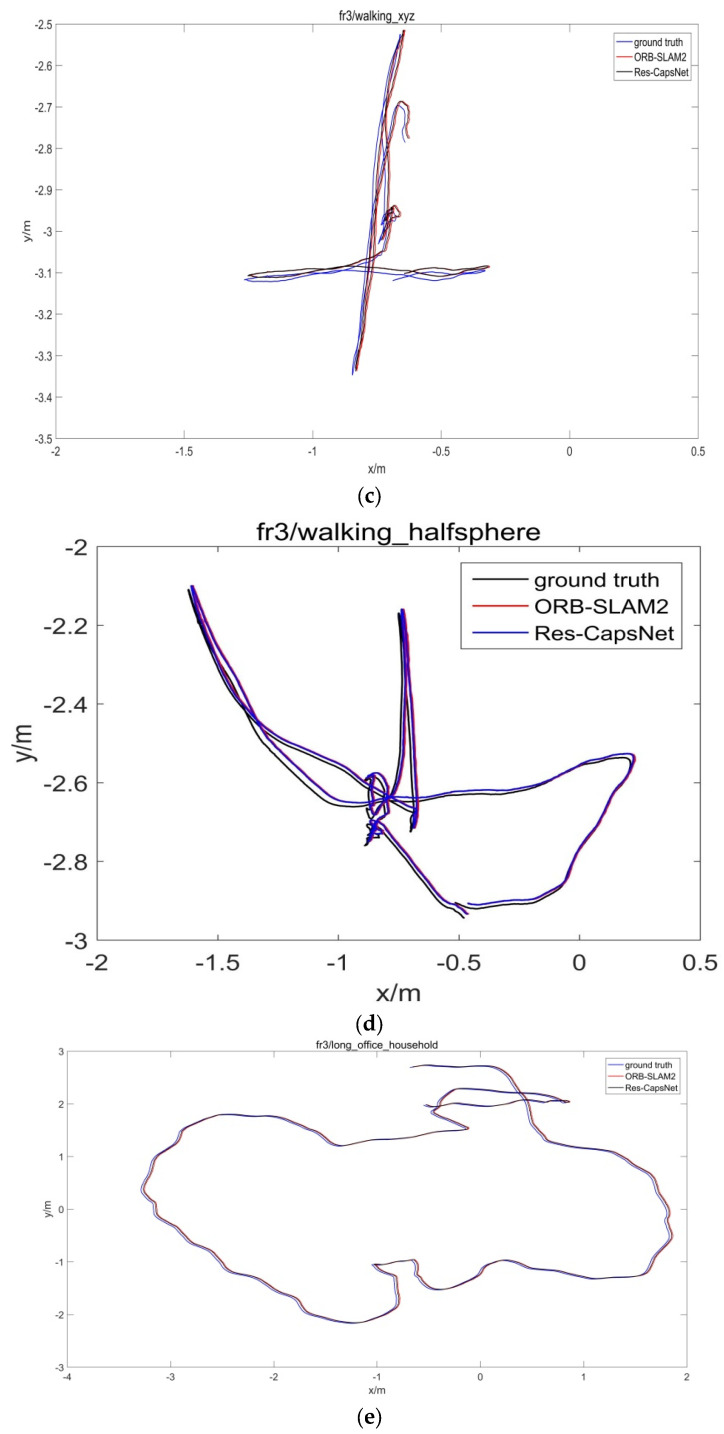

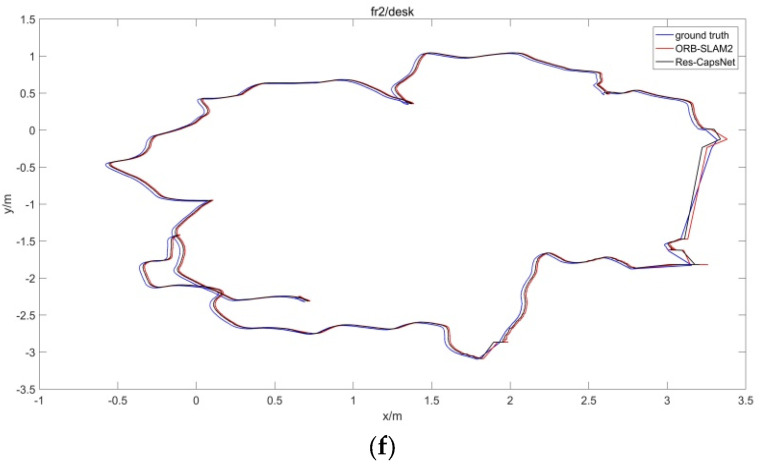
Absolute trajectory error comparison for SLAM. (**a**) fr3/sitting-xyz, (**b**) fr3/sitting-halfsphere, (**c**) fr3/walking-xyz, (**d**) fr3/walking-halfsphere, (**e**) fr3/long-office, (**f**) fr2/desk.

The TUM dataset provided the actual camera pose, and the accuracy of pose estimation for the SLAM system was evaluated by comparing the estimated pose with the real pose. Figure 15 is the comparison of the estimated trajectory and real trajectory for the TUM dataset. The black curve denotes the real trajectory, the red curve denotes the ORB-SLAM2-estimated trajectory, and the blue curve denotes the Res-CapsNet-estimated trajectory.

Figure 15 shows the SLAM system trajectory comparison results of Res-CapsNet and ORB-SLAM2 in low-dynamic, high-dynamic, and large-scale scenarios. The trajectory estimation results of the Res-CapsNet and classical ORB-SLAM2 methods were close to the real trajectory in the low-dynamic scenarios, as shown in Figure 15a,b. The results show that both Res-CapsNet and ORB-SLAM2 have good localization accuracy in low-dynamic scenarios. ORB-SLAM2 has a large error in trajectory estimation in a high-dynamic scene, as shown in Figure 15c,d. Under the condition of violent camera shaking or rapid movement, ORB-SLAM2 cannot accurately distinguish static or dynamic features in the scene, and the accuracy of pose estimation is reduced due to the influence of dynamic features. The estimated trajectory of Res-CapsNet was closer to the real trajectory, with a higher accuracy. Compared with ORB-SLAM2, Res-CapsNet maintained a higher accuracy in large-scale scenarios, as shown in Figure 15e,f. To sum up, Res-CapsNet maintains high accuracy and robustness in complex scenarios.

Table 3 shows the absolute trajectory error (ATE) results of ORB-SLAM2 and Res-CapsNet between the estimated trajectory and the real trajectory in the TUM dataset. Since the RANSAC algorithm of ORB-SLAM2 can eliminate the interference of outside-point motion in low-dynamic scenes, the accuracy of ORB-SLAM2 and Res-CapsNet were similar, and the performance of SLAM was not significantly improved. Compared with ORB-SLAM2, the ATE of Res-CapsNet decreased significantly in high-dynamic and large-scale scenarios. The Res-CapsNet method improved performance by 72.68%, 60.73%, 20.88%, and 27.91%, respectively, in the fr3/walking-xyz, fr3/walking-halfsphere, fr3/long-office, and fr2/desk sequences. This shows that the SLAM based on Res-CapsNet has higher localization accuracy and better robustness in complex scenarios.

Table 4 shows the time consumption of the feature extraction algorithms. The feature extraction time of Res-CpasNet was lower than the ResNet and VGG16 methods and higher than the AlexNet and Faster R-CNN methods. The real-time performance of Res-CapsNet can guarantee the real-time requirement of the SLAM system in complex scenarios.

## 5. Conclusions

We proposed a loop closure detection method based on optimized ResNet and CapsNet. ResNet was used to extract the deep features of images, and GhostVLAD feature coding was introduced to achieve image feature clustering, which solves the problems of network gradient disappearance and network degradation and improves the network convergence speed. The optimal truncation distance was solved by optimizing the minimum value of entropy, the dynamic routing mechanism of the capsule network was improved by using the peak value of entropy density, and the relative spatial location information between features was extracted. Combined with global feature descriptors and feature vectors extracted from CapsNet, the deep network’s ability to recognize and describe image features was improved, and the differences and correlations among features were retained, thus improving the overall performance of the network. The experimental results show that the average accuracy of Res-CapsNet is the highest, which effectively realizes the loop closure detection of a mobile robot in complex scenes, such as illumination changes, view changes, weather changes, and dynamic and large-scale scenes; reduces the cumulative error of the visual odometer; realizes the establishment of a global consistent environment map; and improves the accuracy and robustness of mobile robot SLAM.

## Figures and Tables

**Table 1:** The Gardens Point dataset.

Environmental Changes	Compare the Subsequence	Subsequence 1	Subsequence 2
Illumination changes	day-right vs. night-right (Fig.126-GP)	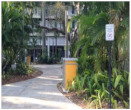	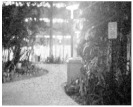
View changes	day-left vs. day-right (Fig.105-GP)	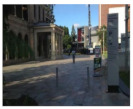	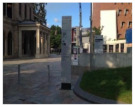
Dynamic environment occlusion	day-right vs. night-right (Fig.103-GP)	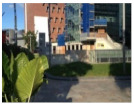	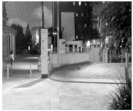
Dynamic environment pedestrian	day-right vs. night-right (Fig.56-GP)	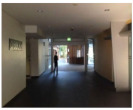	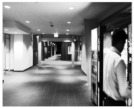

**Table 2:** The parameters of TUM dataset.

Dataset Attributes	Dataset	Duration (s)	Average Speed of Movement (m/s)	Mean Angular Velocity (deg/s)	True Trajectory Length (m)
Low-dynamic	fr3/sitting-xyz	42.50	0.132	3.562	5.496
	fr3/sitting-halfsphere	37.15	0.180	19.094	6.503
High-dynamic	fr3/walking-xyz	28.83	0.208	5.490	5.791
	fr3/walking-halfsphere	35.81	0.221	18.267	7.686
Large-scale	fr3/long-office	87.09	0.249	10.188	21.455
	fr2/desk	99.36	0.193	6.338	18.880

**Table 3:** Comparison of absolute trajectory error.

Sequence	ORB-SLAM2/(cm)	Our Method/(cm)	Performance Improvement
fr3/sitting-xyz	0.95	0.87	8.42%
fr3/sitting-halfsphere	7.75	6.62	14.58%
fr3/walking-xyz	73.80	20.16	72.68%
fr3/walking-halfsphere	49.93	19.61	60.73%
fr3/long-office	10.44	8.26	20.88%
fr2/desk	0.86	0.62	27.91%

**Table 4:** Time consumption of feature extraction algorithms.

Feature Extraction Algorithms	Network Models	Dataset Tasks	Calculation Rate (ms/Frame)
AlexNet-CONV3	AlexNet [42]		167
AlexNet-POOL5			147
VGG-M1024	VGG-M1024 [43]	ImageNet classification [44]	249
VGG16	VGG16 [20]		1379
FastRCNN-CaffeNet	Fast R-CNN [45]	PASCAL VOC	23
FastRCNN-VGG-M1024			36
FastRCNN-VGG			128
Faster RCNN-ZF	Faster R-CNN [46]	Object detection [47]	49
Faster RCNN- VGG16			157
SqueezeNet	SqueezeNet [48]	ImageNet classification [44]	245
ResNet	ResNet [25]		1517
Res-CapsNet	Res-CapsNet	SLAM [41]	256

## Data Availability

Not applicable.
